# Parasitological, serological and molecular diagnosis of acute and chronic Chagas disease: from field to laboratory

**DOI:** 10.1590/0074-02760200444

**Published:** 2022-06-01

**Authors:** Alejandro Gabriel Schijman, Julio Alonso-Padilla, Silvia Andrea Longhi, Albert Picado

**Affiliations:** 1 Instituto de Investigaciones en Ingeniería Genética y Biología Molecular Dr Hector Torres, CONICET, Laboratorio de Biología Molecular de la Enfermedad de Chagas, Ciudad de Buenos Aires, Argentina; 2 Barcelona Institute for Global Health, University of Barcelona, Hospital Clinic, Barcelona, Spain; 3 Foundation for Innovative New Diagnostics, Geneva, Switzerland

**Keywords:** Chagas disease, Trypanosoma cruzi, diagnosis, polymerase chain reaction, loop mediated isothermal amplification

## Abstract

There is no consensus on the diagnostic algorithms for many scenarios of *Trypanosoma cruzi* infection, which hinders the establishment of governmental guidelines in endemic and non-endemic countries. In the acute phase, parasitological methods are currently employed, and standardised surrogate molecular tests are being introduced to provide higher sensitivity and less operator-dependence. In the chronic phase, IgG-based serological assays are currently used, but if a single assay does not reach the required accuracy, PAHO/WHO recommends at least two immunological tests with different technical principles. Specific algorithms are applied to diagnose congenital infection, screen blood and organ donors or conduct epidemiological surveys. Detecting Chagas disease reactivation in immunosuppressed individuals is an area of increasing interest. Due to its neglect, enhancing access to diagnosis of patients at risk of suffering *T. cruzi* infection should be a priority at national and regional levels.

Chagas disease (CD), caused by *Trypanosoma cruzi*, is most likely “the most neglected of the neglected diseases”.^1^ It has been treated as an endemic disease in tropical and subtropical areas of South, Central America, Mexico and Southern United States.^2^ Chagas disease is also an emerging global concern in non-endemic areas.^3^

Once vectorial and transfusional control have been achieved, perpetuation of infection occurs mainly through congenital transmission in endemic and non-endemic areas.^4^ Mother-to-child transmission of *T. cruzi* has repercussions in terms of individual and global public health: 1.12 million women of childbearing age are infected, and ~ 9,000 infected babies are born each year, accounting for around 22% of all new cases of CD.^5^ ^,^ ^6^ In non-endemic countries, congenital infection is the first cause of new cases of CD.^5^ ^,^ ^7^ Since *T. cruzi* maternal-foetal transmission can be repeated at each pregnancy and observed from one generation to another, this way of transmission can easily extend in time.^8^ Consequently, interruption of this form of transmission by expanding access to diagnosis and treatment in target populations is one of the main 2030 targets of the WHO road map.

In rural zones, outbreaks of oral infection are becoming more frequent. It is considered the main route of infection in the Brazilian Amazon and in Venezuela, with reports in other Latin American countries, such as Colombia, Bolivian Amazon, Brazil and French Guiana.^9^ ^,^ ^10^ ^,^ ^11^

Moreover, research personnel working in *in vitro* or experimental models of infection, immunisation and treatment should take special care in manipulations to avoid accidental contamination with the parasite, by means of good laboratory practices and use of personal protection equipment.^12^

The infection passes through an acute phase, evolving to an asymptomatic or symptomatic chronic phase, with different degrees of progression and severity.^13^ The acute phase is characterised by high parasitaemia, with detectable parasites in blood. Nevertheless, most cases remain undetected because symptoms are usually insignificant and non-specific, and health care personnel may not suspect *T. cruzi* infection. In general, the acute phase resolves with a decrease in parasite burden a month after primary infection.^13^ Thus, the majority of *T. cruzi* acute infections progresses onto a silent chronic phase, also named indeterminate or asymptomatic chronic Chagas disease, throughout which parasitaemia is low and intermittent. As most asymptomatic chronic CD people are unaware of their infection status, they may only be diagnosed when donating blood or submitted to a health check.

In non-endemic regions receiving migration from Latin-America, physicians should be aware of the potential occurrence of cases of imported CD or cases of congenital transmission in children born to infected mothers. Accordingly, proper laboratory tests should be requested to confirm or dismiss *T. cruzi* infection.^3^

Ten to forty percent of individuals chronically infected by *T. cruzi* will develop clinical signs and symptoms of CD years after getting infected. This entails a high lifetime risk of presenting severe cardiac complications (cardiomegaly, complex arrhythmias or heart failure), intestinal disorders (megacolon, megaesophagus) or cardiodigestive presentations. In CD endemic areas, more than 90% of patients presenting one of these clinical manifestations are infected with *T. cruzi*.^12^

The different phases of the disease and modes of transmission determine which diagnostic strategies or tests should be used. Indeed, different diagnostic assays should be applied during epidemiological surveys, surveillance for vector-borne transmission, blood bank screening, screening of pregnant women and their offspring to assess vertical transmission, and for the diagnosis of chronic CD. Additionally, access to diagnosis (and treatment) should be improved, especially in CD-endemic regions where diagnostic laboratories are scarce and ill-equipped. The use of point-of-care (POC) diagnostics in those regions would help reaching more patients.^14^ ^,^ ^15^

**PARASITE DIVERSITY AND DIAGNOSIS**

The genetic structure of *T. cruzi* is mainly a consequence of clonal propagation with extraordinary events of genomic exchange.^16^ ^,^ ^17^ Biological, biochemical and molecular markers have demonstrated vast genetic polymorphism.^18^ ^,^ ^19^ At the moment, natural populations of the parasite are classified into six discrete typing units (DTUs TcI to TcVI), composed of sets of stocks genetically closer to one another than to any other one.^20^ ^,^ ^21^ In addition, *T. cruzi* bat (Tcbat) has been proposed as an independent DTU infecting fruit-eating bats.^22^ All DTUs I to VI are causative of CD^20^ and at present, a single human case report of *T. cruzi* infection has been attributed to Tcbat.^23^ The DTUs are identifiable by specific molecular and biochemical markers and exhibit particular geographic distribution. They harbour different DNA content and gene dosage^24^ and may have preferential tropism for vector and reservoir species, as well as tissue tropism within an infected host, as a reflection of the varied phenotypic presentation of their genetic diversity. Even within the same DTU, important genetic heterogeneity among different strains exists, which has been associated with, linked to different histological tropism and virulence, in turn linked to different clinical manifestations as well as drug susceptibility.^19^ ^,^ ^24^ ^,^ ^25^ ^,^ ^26^ ^,^ ^27^ ^,^ ^28^ This vast genetic diversity should be considered when developing diagnostic tests, especially if assays are intended for worldwide use. Any novel diagnostic assay should be validated with strains representing at least the DTUs prevailing in the region where this test is to be applied. Furthermore, some molecular targets used to develop diagnostic assays based on recombinant antigen or nucleic acid amplification strategies, are polymorphic and present dissimilar gene copy numbers and levels of gene expression in different strains belonging to different DTUs, and in some instances also in strains from the same DTU.

**Diagnostics strategies in different**

**Chagas disease settings**

Table I outlines the diagnostic methodologies used for the different scenarios of CD, described in the following sections.

**TABLE I:** Main methods for diagnosis of *Trypanosoma cruzi* infections according to the phase of the disease and transmission routes

Epidemiological settings	Parasitological methods			Molecular methods		Serological methods						
	Direct microscopic observation	Indirect observation		qPCR	LAMP	ELISA		IHA	IIF	TESA-blot	CMIA	Rapid diagnostic tests
		Haemoculture	Xenodiagnosis			Whole lysate^*a*^	Recombinant proteins/ peptides					
Vector transmission	Strout/ wet smear											
			
Congenital transmission	MH^*b*^ / Micro Strout											
	
Oral transmission	Strout/ wet smear											
			
Transfusion transmission	Strout/ wet smear											
Seronegative receptor of organ from seropositive donor	Strout											
Reactivation by immunosuppresion	Strout											
Blood bank												

*a*: cross reaction with *Trypanosoma rangeli* and *Leishmania* spp.; *b*: microhematocrite. Darker grey boxes: acute Chagas disease (CD); lighter grey boxes: chronic CD. CMIA: chemiluminescent magnetic immunoassays; ELISA: enzyme-linked immunosorbent assay; IHA: indirect haemagglutination assay; IIF: indirect immunofluorescence; LAMP: loop mediated isothermal amplification; qPCR: quantitative polymerase chain reaction; TESA-blot: trypomastigote excreted-secreted antigens-blot.

*Epidemiological surveys* are essential to diagnose *T. cruzi-*infected people from endemic areas. For operational reasons, epidemiological surveys seek to collect the maximum number of samples per day. Different strategies are used to increase the number of people screened. The use of filter paper to collect blood simplifies the sample collection but still requires transferring the samples to a laboratory for testing.

Serological methods are also used in epidemiological surveys. Samples with an indeterminate result or low-level reactivity present challenges when estimating prevalence or incidence rates for epidemiological surveillance.

Rapid diagnostic tests (RDTs) have been proposed for epidemiological surveillance (Table I). Nevertheless, according to the current guidelines,^29^ RDT positive individuals still need to be confirmed by a second laboratory test before treatment can be started, limiting the benefit of these tools.^15^ ^,^ ^30^ Recent studies have shown that, in highly endemic regions, combining two RDTs could allow diagnosing *T. cruzi* infected individuals in the field with the same accuracy as that provided by performing two laboratory-based tests.^15^ ^,^ ^31^ ^,^ ^32^

**Diagnosis of acute phase infections**

Independently of its source of acquisition, the acute phase is defined by the presence of patent parasitaemia.

*Vector-borne transmission* was the first mechanism of infection described in the history of CD.^33^ It presents an incubation period of 1-2 weeks. Signs of portal of entry, namely indurated cutaneous lesion (chagoma) or palpebral edema (Romaña sign) are detectable in a minority of cases. Most cases are accompanied by mild symptomatology (95-99%) and go unrecognised. However, persistent fever, fatigue, lymphadenopathy, hepatomegaly, splenomegaly, morbilliform rash and edema can occur. In rare cases, myocarditis or meningoencephalitis, anemia, lymphocytosis, raised aspartate transaminase (AST) and alanine aminotransferase (ALT) concentrations are observed, which entails a risk of mortality around 2-5%.

Due to the variability and non-specificity of the symptoms, to aid in the diagnosis, physicians in endemic areas should complement the clinical findings with epidemiological data, e.g., CD cases already diagnosed in the same locality and triatomines found in the domiciles or peridomiciles.

After an incubation period of some days, the circulating parasites are detectable by direct microscopic observation. In its simplest format, the wet smear consists in searching for motile trypomastigotes in a drop of blood, placed between a slide and a coverslip. With the aid of 40X objective lens with reduced condenser aperture, or by recognising them among red blood cells by phase-contrast, the parasites can be detected. When trypomastigotes are not observed and clinical suspicion persists, a concentration method may be applied to increase sensitivity.^34^ The method of Strout requires 2 to 5 mL of venous blood without anticoagulants.^35^ Once the clot is formed, the liquid phase is transferred to a test tube and spun down for 5 min at 50 to 100 g. The supernatant is then transferred to another tube and spun down at 400 g to allow parasites to decant to the bottom of the tube. The supernatant is discarded and the last drop mounted on a slide using the same procedure as for the wet smear. As in other procedures of microscopic observation, the sensitivity of this method is highly dependent on the operator’s expertise and available working time to dedicate to the examination of the sample, which tend to be limited in health facilities serving CD endemic communities.

Indirect methods are based on the proliferation of parasites on animals or *in vitro* culture systems. Historically, xenodiagnosis was the first procedure used when the disease was described. Its rationale is to feed triatomine bugs with the patients’ blood, and 30 to 60 days after being fed, examine their feces for the presence of parasites. Usually, four boxes with ten bugs each are utilised in the diagnostic procedure. Traditionally, a box containing triatomine bugs was placed onto the tested persons’ arms and legs, but nowadays heparinised blood collected from the tested person is given to the bugs through a latex membrane (artificial xenodiagnosis).^36^ Nonetheless, xenodiagnosis is rarely used as it can only be performed in referral centres where triatomines are bred.

Haemoculture is based on harvesting heparinised blood (i.e., 20 mL) from a suspected case, removing the plasma, and adding sterile media to support the growth of any potential bloodstream trypomastigote present in the sample. Examination of the culture should be performed monthly for as long as six months to reach a diagnosis.^37^ Mice inoculation with patients’ blood or with feces from bugs after xenodiagnosis is also possible but seldom employed. In this case, tail blood of inoculated mice should be examined daily for 1 to 2 months.^38^

However, sensitivity of these indirect parasitological methods is low and variable (around 20%), being highly dependent on the operator skills and experience. If the method is repeated, the probability of detection increases (up to 60% of sensitivity) but for some patients with very low parasitaemia even successive examinations will be negative.^39^

If the parasite is difficult to find during the acute phase, the search of an anti-*T. cruzi-*specific IgM response can be applied. Among the few serological methods that have been developed for the diagnosis of acute CD IgM type humoral response against the shed acute phase antigen (SAPA), member of the *trans*-sialidase family, has been most investigated.^40^ Search for anti-*T. cruzi* IgM antibodies may be performed when an acute case is suspected and parasites are not found. SAPA is an “in house” test, not commercially available, which may give rise to false positive results when rheumatoid factor is present.^41^ Another difficulty for performing IgM-based serodiagnosis is the lack of serum specimens from acute phase patients to be used as proper positive controls.^12^ In the clinical practice, with the introduction of molecular techniques, IgM-based strategies and indirect parasitological methods are gradually being disused.

*Congenital transmission* occurs via transplacental infection during pregnancy and a low proportion of cases may become infected at labor.^42^ Although most (around 60%) congenitally infected newborns are asymptomatic at birth, they display higher frequencies of low Apgar scores, low birth weight and prematurity than uninfected newborns, and some suffer from severe symptoms that can rapidly lead to death.^43^ ^,^ ^44^ Congenitally infected infants are at risk of developing, years later, disabling and life-threatening chronic pathology.^42^ ^,^ ^45^ ^,^ ^46^ ^,^ ^47^ Therefore, it is of utmost importance to prevent congenital transmission as well as to rapidly diagnose and treat congenitally infected newborns. This must be particularly stressed, considering that the administration of treatment close to delivery achieves cure rates of almost 100%.^42^

The first step to control vertical transmission is to diagnose *T. cruzi*-infected pregnant women. Once confirmed, their offspring must be tested for *T. cruzi* infection. In women who give birth in maternity services, it is ideal to have a parasitological/molecular diagnosis of the newborns within their first 72 h after delivery, to enable referring them to treatment in case of positive findings and to avoid loss to follow-up after the mother-baby binomial leave the hospital. 

The current diagnostic algorithm of congenital infection encompasses several steps (Figure). Parasitological diagnosis must be performed at birth or during the first months of life. It needs a fresh sample to enable detection of motile trypomastigote forms. These procedures require trained laboratory personnel, which is often not the case in endemic areas. Success of diagnosis is variable depending on the operator skills and sensitivity is low. Therefore, babies negative to the parasitology test must be tested by a serological assay at 9 to 12 months of age, when maternal antibodies wane. In many endemic regions, affected people live far from referral health centres and afford to travel, which limits access to a definite diagnosis in a large proportion of infants. Loss to follow-up results in patients not being treated and evolving to the chronic phase of the disease and potentially developing the life-threatening symptomatology several years later.

**Figure:**
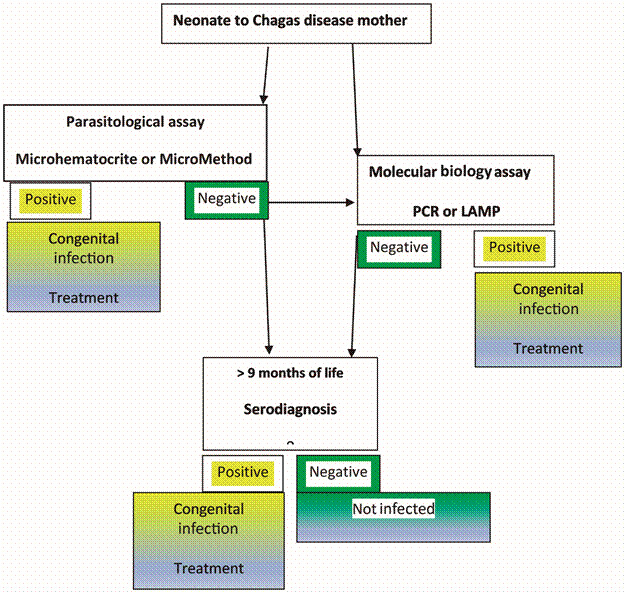
Diagnostics algorithm for congenital Chagas disease.

In newborns or neonates, who have low blood volume, the method of microhematocrite should be used by filling up to 4-6 capillary tubes and, after spinning them, looking on the interface between red blood cells and plasma under the microscope. The capillary tubes are broken at the interface and processed as described earlier with the wet smear.^48^ A modification of the microhematocrite is the micromethod, which uses microtubes instead of capillary tubes, thus reducing the potential harm to the operator.^49^ As mentioned above, the sensitivity of the microhematocrite or micromethod largely depends on the operator’s experience and time available to observe each sample, reaching an average sensitivity of around 50% in public health institutions.

To fill this gap, molecular methods in newborns or infants have been tested for sensitive early diagnosis to avoid loss to follow-up.^50^ ^,^ ^51^ ^,^ ^52^ ^,^ ^53^ ^,^ ^54^ A TaqMan real-time polymerase chain reaction (qPCR) based kit, including an internal control of DNA integrity of the sample or reaction inhibition, has achieved higher sensitivity than the micromethod starting from 1 mL of peripheral blood mixed with a DNA stabiliser solution.^54^ Studies in infants born to seropositive mothers have observed that the best age to carry out molecular diagnosis of congenital transmission is around the first month of life, when the parasitic load is at its peak,^49^ ^,^ ^55^ and potential false positive results that might arise from the transmission of *T. cruzi* DNA from the mother to the fetus are minimised.^42^

Loop mediated isothermal amplification (LAMP), is an alternative molecular approach, more suitable to resource-limited laboratories, because the strand-displacement-*Bst* DNA polymerase works at 60-65ºC and does not require of a thermocycler, but only a thermoblock or water bath.^56^ ^,^ ^57^ Furthermore, product visualisation can be done by the naked eye or followed in real time by turbidity or fluorescence. LAMP procedures have been proposed for detection of *T. cruzi* infection.^53^ ^,^ ^58^ A prototype kit based on *T. cruzi* satellite DNA sequences and containing dried reagents on the inside of the microtube caps has proved to be as sensitive as qPCR in blood samples from congenitally infected cases.^53^ ^,^ ^59^ Table II summarises standardised in-house and commercial molecular-based methods for detection of *T. cruzi* infection.

**TABLE II:** Primers and TaqMan probes sequences for *Trypanosoma cruzi* validated quantitative polymerase chain reaction (qPCR)

Molecular target	Methodology	Primer/Probe sequences (5’-3’) or Kit company/Consortium	Reference
*T. cruzi* satellite DNA	Conventional PCR or real time PCR	TcZ-F “GCTCTTGCCCACAMGGGTGC”	82
		Tcz-R “CCAAGCAGCGGATAGTTCAGG”	
	Standardised in-house TaqMan qPCR	Cruzi 1 “ASTCGGCTGATCGTTTTCGA”	82,85
		Cruzi 2 “AATTCCTCCAAGCAGCGGATA”	
		Cruzi 3 Probe “FAM-CACACACTGGACACCAA-NFQ-MGB”	
	Comercial real time PCR	Wiener Lab-CONICET-ANLIS MALBRAN-Argentina	54
		RealCycler CHAG; Progenie Molecular, Spain	60
		TCRUZI DNA.CE Diagnostic Bioprobes Srl, Italy	61
	Comercial LAMP	Eiken Chemical Company, Japan	53,59
*T. cruzi* minicircle DNA	Conventional PCR	121 “*AAATAATGTACGGGKGAGATGCATGA*”	50,51,52,82
		122 “GGTTCGATTGGGGTTGGTGTAATATA”	
	Standardised in-house TaqMan qPCR	32F “TTTGGGAGGGGCGTTCA”	85
		148R “ATATTACACCAACCCCAATCGAA”	
		71P Probe “FAM-CATCTCACCCGTACATT-BHQ1^*a*^ ”	
	Comercial real time PCR	Real STAR Chagas, ALTONA Diagnostics, Germany	62

*a*: C locked nucleic acid (LNA) nucleotides.

Other means of diagnosis, such as determination of IgM reactivity in congenital cases has been proposed,^63^ ^,^ ^64^ ^,^ ^65^ although it is not employed in clinical practice. An antigen detection assay based on the use of nanoparticles (Chunap) has been developed for diagnosis of congenital CD in a single urine specimen at one month of life with more than 90% sensitivity and more than 95% specificity.^66^ However, an ultra-centrifuge would be required to perform it, and thus it would be unfeasible in many microbiology laboratories.

*Oral transmission* outbreaks have been recognised as a frequent mechanism of infection over the last decade, mostly impacting rural and sylvatic environments or urban locations bordered by wild areas with triatomine populations.^9^ ^,^ ^10^ ^,^ ^11^ ^,^ ^67^ ^,^ ^68^ ^,^ ^69^ Such outbreaks may involve many individuals, usually within the same family/school or after social events, causing micro-epidemics.^9^ ^,^ ^70^ Indeed, observing several related individuals with fever and sometimes cardiac manifestations, digestive involvement, abdominal pain, jaundice in some cases, may suggest the occurrence of foodborne transmitted outbreaks. This transmission route is highly efficient due to the high parasitic burden in the ingested meal, and lethal cases are more frequent than those caused by vector-borne transmission. In most outbreaks, molecular methods have been critical for specific and rapid diagnosis as well as to identify the source of the infection by genotyping the parasite strain involved. In addition, molecular methods have been useful to confirm treatment failure that has been observed in a large proportion of orally infected patients.^71^

*Blood transfusion transmission*, same as the congenital and organ transplant routes, is of relevance in endemic and non-endemic regions alike. Globalisation, with increased travel and immigration, presents a risk of exposure to infectious agents and represents a global problem for blood banks. Because CD is endemic in Latin America, the strategy to prevent transmission of blood transfusion infections is to identify specific groups of donors, migrants or travelers, from endemic areas using questionnaires during pre-donation procedures. Then, additional determinations, such as the use of two different serological tests, further reduce the risk of transmission.^72^ ^,^ ^73^ However, only around 20% of blood donors with asymptomatic chronic CD have been reported to transmit the parasite to seronegative receptors, probably due to the paucity of trypomastigotes.^12^

In acute cases of transfusional CD, high levels of parasitaemia usually occur, but as the receptor had to be transfused because of a disease other than Chagas, *T. cruzi* infection is not suspected and it is more likely detected in stained smears carried out to count leukocytes. Acute CD may present symptoms only many days after the transfusion event.

*T. cruzi transmission by organ transplantation* in naïve receptors is exacerbated due to the administration of immunosuppressive therapy.^74^ A special situation is the acute phase that may emerge in seronegative recipients of organs from seropositive donors. In general, Strout is used, but molecular diagnosis may be particularly useful to detect infection earlier. For instance, PCR enabled the detection of bloodstream *T. cruzi* DNA between 28 and 47 days earlier than Strout,^75^ and the LAMP technology has also shown its potential to follow-up these cases.^59^

*Chagas disease reactivation* in chronically infected patients that acquire HIV or receive immunosuppressing therapies after organ transplantation, autoimmune diseases or cancer, usually entails parasitaemia increases leading to an acute disease known as CD reactivation.^74^ ^,^ ^76^ ^,^ ^77^ Since the patient is already infected, anti-*T. cruzi* IgGs are also detectable. Thus, exclusion of *T. cruzi* infection by serology should be mandatory in these cases. Due to low numbers of CD4 T cells in patients also suffering from AIDS, and immunosupression from long-term drug use, parasitaemias are generally high in these cases, with frequent complications, including high-morbidity such as panniculitis, acute myocarditis and meningoencephalitis.

Routine follow-up of these patients is also carried out using Strout, but stained smears are less sensitive and only appropriate with high parasitaemia levels, which may be observed when clinical manifestations of reactivation are detectable. In cases of immunosuppression resulting from heart transplantation, reactivation has been anticipated by means of molecular methods in peripheral blood and endomyocardial biopsy samples.^78^ ^,^ ^79^ ^,^ ^80^ Again, *T. cruzi*-LAMP has also proved useful to detect parasite presence in samples from organ-transplanted patients showing high agreement with real-time PCR findings.^59^

In HIV coinfected CD patients, molecular methods have been useful for differential diagnosis of meningoencephalitis caused by *T. cruzi* or toxoplasmosis, starting from brain chagoma biopsies or cerebrospinal fluid samples, allowing prompt specific therapy decisions.^25^ LAMP appears also helpful to detect *T. cruzi* DNA in bloodstream or cerebrospinal fluid samples in these cases.^59^ Additionally, Chagas urine nanoparticle test (Chunap) also showed potential for early detection of Chagas reactivation in *T. cruzi*/HIV patients.^81^

**Diagnosis of chronic phase**

In the chronic phase, diagnosis is performed with IgG-based serological tests. In chronic CD patients, molecular-based detection methods have a limited diagnosis value, as they have significantly lower sensitivity than serology-based tests.^82^ ^,^ ^83^ This may be related to low and variable bloodstream parasitaemia levels.^84^ ^,^ ^85^ Most of the assays employed during the last 40 years for the diagnosis of chronic CD are conventional serological tests to detect anti-*T. cruzi* IgG levels; namely: indirect haemagglutination assay (IHA), enzyme-linked immunosorbent assay (ELISA) and indirect immunofluorescence (IIF).

IHA is the simplest and less expensive assay. The procedure has few steps, which reduce manipulation errors. Sensitised red blood cells from an animal species (generally sheep) and serum from the patient are placed in contact for 1 or 2 h. After this time, if parasite-specific antibodies are present in the sera, the red blood cells make a net on the bottom of the tube or well, which is read by naked eye. If the red blood cell sediment on the bottom is a point, the reaction is interpreted as negative. Serial dilutions permit to estimate the titer of the reaction.

ELISA includes the contact of the patients’ serum with antigens of the parasite attached to the treated plastic material of a microplate well. These antigens can be inactivated whole parasite lysates, which can lead to cross-reactivity with antigens of other trypanosomatids like *Leishmania* spp., or recombinantly engineered proteins or their specific moieties (epitopes) selected to minimise the event of such cross-reactivity while preserving high sensitivity and specificity ratios.

The IIF needs of a fluorescent microscope, demand several incubation steps, being thus time-consuming, and the interpretation of results is operator dependent. Its main advantage is its high sensitivity (> 99%) but specificity is not as good (> 96%) especially because of cross-reactivity with several diseases. This cross-reactivity is mainly detected in samples exhibiting low titers (1/40 to 1/80).^12^

For all these conventional tests, results obtained may be non-reactive (negative), reactive (positive) or borderline (gray zone), and two of them must be concordantly positive or negative to guarantee the confidence of results.

Non-conventional serological tests based on different principles have also been employed for the diagnosis of chronic CD. These were developed after the discovery and validation of immunogenic *T. cruzi* antigenic families. Genetic engineering strategies have achieved the construction of recombinant antigens suitable to different immunological assay formats, such as mixture of recombinant antigens,^86^ short peptides^87^ or antigenic matrices based on chimeric proteins composed of repetitive and conserved immunodominant amino acid fragments of several *T. cruzi* proteins.^88^

Such recombinant antigens have been used in last generation ELISA tests that have higher sensitivity and specificity than assays based on whole lysate antigens (Tables I, III). Alternatively, recombinant immunological assays can use high sensitivity detection formats, with chemiluminescence being the most employed. This assay format is commercially available at a high cost and used in many blood banks and some clinical laboratories. Its sensitivity is around 100% and its specificity is also remarkably high, which prompted some authors to suggest it could be used as a single diagnostic.^89^ Only gray zone and weak positives giving ≤ 6 signal over cut off values should be evaluated with a second conventional immunological test.

**TABLE III:** *Trypanosoma cruzi* recombinant proteins/synthetic peptides for rapid diagnostics tests and epidemiological studies

Applications	Antigen name	Corresponding protein
Acute / congenital infections	SAPA / TCNA / TS	Trans-sialidases family
Acute /Chronic infections	Ag36 / JL9 / MAP	Microtubule-associated protein
Chronic infection	B13 / Ag2 / TCR39 / PEP2 / TSSA	Trypomastigote surface protein
	CRA / Ag30 / JL8 / TCR27	Cytoplasmatic antigen
	FRA / Ag1 / JL7 / H49	Cytoskeleton-associated protein
	FcaBP / 1F8 / Tc-24 / Tc-28	Flagellar Ca++ binding protein
	TcE / JL5 / TcP0	Ribosomal proteins
	cy-hsp70 / mt-hsp70 / grp-hsp78	Heat shock proteins
	FL160 / CEA / CRP	Flagellum-associated surface protein protein
	SAPA, Ag 1, 2, 13, 30, 36, KMP11, HSP70, PFR2, Tgp63	Antigens used in grouped panels

CRA: cytoplasmatic repetitive antigen; FRA: flagellar repetitive antigen; MAP: microtubule associated protein; SAPA: shed acute-phase antigen; CEA: chronic axoantigen; CRP: complement regulatory protein; cy-hsp: cytoplasmatic heat shock protein; grp-hsp: endoplasmatic reticulum heat shock protein; mt-hsp: mitochondrion heat shock protein; TCNA: *Trypanosoma cruzi* neuraminidase; TS: trans-sialidase; TSSA: trypomastigote small surface antigen. Several different antigen names given to identical or similar recombinant antigens or peptides are grouped as x/y/z.

Also based on chemiluminescent readout is the immunoassay based on glycan-rich antigens containing the terminal determinant α-galactopyranosyl (*α-Gal*). Such epitopes are highly abundant on the surface of trypomastigote forms as part of *O*-glycans,^90^ and their use as diagnostic biomarkers has been evaluated in cohorts of CD patients.^91^ ^,^ ^92^ ^,^ ^93^

Other non-conventional tests are lytic assays, including flow cytometry and RIPA (radioimmunoassay), which are not commercially available.^12^

Western blot or Dot blot tests are also used to confirm infection by means of discrete recognition of specific antigens. The trypomastigote excreted secreted antigens (TESA) blot is used in some countries of Latin America for serodiagnosis confirmation.^94^ The Food and Drug Administration (FDA) has approved the Abbott ESA Chagas Dot blot assay, which harbours the same recombinant antigens as the Abbott ARCHITECT screening method.^95^ In addition, a multiplex ELISA-based array that includes 12 different antigens printed on 96-well plates has been developed for confirmation of infection. Interestingly, the sum of all antigens was shown to reflect the level of parasitaemia detected by PCR;^96^ but its clinical validation is still required on a large set of samples.

The current WHO/PAHO recommendation is to use at least two serological tests based on different technical principles to avoid false results.^97^ The titer of each reaction should be included so the possibility of errors with high titers is minimised. Each laboratory should include a table with the non-reactive values, those in the gray region, and the positive values, which may differ from laboratory to laboratory.

*RDTs* are valuable alternatives for remote regions, where delays in processing ELISA tests and returning the results hinder diagnosis of chronic *T. cruzi* infections. RDTs are immune-chromatographic assays like those used to diagnose other conditions such as pregnancy, HIV, malaria and others. In the case of tests to detect *T. cruzi* infections, a membrane is sensitised with several recombinant antigens and a small volume of serum or whole blood is placed in contact with it. The fact that some RDTs work with a little volume of whole blood, which can be obtained by finger prick, greatly facilitates logistics and patients’ predisposition to be tested. Moreover, they do not require specific equipment for incubation or reading of results, and allow a quick result turnaround between 10 min to less than 1 h, so that the tested person can walk away from the consultation with a result. Several works have been published^98^ ^,^ ^99^ showing reasonable specificity and sensitivity of some of them. Nonetheless, they still have precise indications and should not label an individual as infected unless a second, conventional test is used to confirm the diagnosis. In fact, RDTs are mainly screening tools because current policies still enforce that the diagnosis of the infection must be confirmed by conventional serological methods.^100^ Aiming to overcome this limitation, a combination of two serology-based RDTs has been proposed and tested successfully to arrive at a conclusive CD diagnosis.^15^ ^,^ ^31^ ^,^ ^32^ Nonetheless, RDT performance has been shown to be geographically variable.^101^ ^,^ ^102^

A systematic review evaluated the suitability of using RDTs for the diagnosis of chronic CD in cohorts of selected studies.^103^ Their overall accuracy was 96.6% sensitivity (95% confidence interval (CI): 91.3-98.7%) and 99.3% specificity (95% CI: 98.4-99.7%). The RDTs evaluated showed better accuracy when used in endemic areas with 98.1% of sensitivity and 99.3% of specificity, while in non-endemic areas their sensitivity was about 90%. Among them, Stat-Pak (Chembio, USA) showed highest accuracy, an overall 97% (95% CI: 87.6-99.3) sensitivity and 99.4% (95% CI: 98.6-99.8) specificity, although a comparison between endemic versus non-endemic areas was not possible. Chagas Stat-Pak includes recombinant antigens H49/JL7, 1F8 and B13 (Table III).^98^

Considering that a strategy based on a single RDT would be much easier and cheaper to implement than the classical strategy based on two serological tests, it is very plausible to assume that the few RDT false negative cases would be amply compensated by the possibility of having screened a larger population than if no RDTs were available. This would be especially appreciated in rural areas of endemic countries where access to diagnosis is challenging. Otherwise, all individuals with a positive RDT result should be submitted to a confirmatory test to prevent the side effects of an avoidable treatment. The studies by Eguez and coworkers^15^ and Lozano and co-workers^32^ proposed using two different RDTs to improve screening accuracy. When considering at least one positive out of two RDTs as a criterion to define a *T. cruzi* infected case, the combination reached a near-perfect sensitivity and specificity.

Finally, skin tests (delayed hypersensitivity) and the detection of circulating antigens in serum and in urine have been proposed, but they do not constitute part of routine diagnostics.^104^ ^,^ ^105^

Apart from laboratory tests, the epidemiology and clinical context are relevant for the interpretation of findings. This is particularly important in cases of vis­ceral leishmaniasis (VL) as conventional serology usu­ally gives false positive results in VL cases and the clini­cian should have this in mind.^12^

**Pan American Health Organization guidance for CD diagnosis and treatment**

The Pan American Health Organization (PAHO) developed guidelines for the management of *T. cruzi* infections aiming to strengthen the implementation of public policies and the management of its clinical conditions.^106^ A synthesis of the recommendations stated in the Guide for Diagnosis and Treatment of Chagas Disease, published (in Spanish only) by PAHO in 2018 has been recently published to provide strategies for the timely diagnosis and treatment of CD, as well as considerations for the implementation of such strategies.^106^ These recommendations are applicable to adult and pediatric patients with suspected CD, exposure to *T. cruzi*, or a confirmed diagnosis of acute, chronic, or congenital CD. The recommendations have been classified into two categories: conditional or strong, while the quality of the existing evidence for each recommendation has been classified as high, moderate, low and very low, according to the GRADE approach.^107^ Ten recommendations have been made and five of them are dedicated to diagnosis, namely:

(i) *In patients with suspicion of chronic T. cruzi infection, which is the best diagnosis strategy (one or two serological techniques)?*

In these cases, it is recommended to use the “diagnostics standard”, which is the combination of two serological tests using different antigens capable to detect anti-*T. cruzi* antibodies (ELISA, IHA, IIF), and a third test if the results from such tests are discordant. The quality of the evidence for this recommendation is considered high/moderate.

(ii) *In the framework of seroepidemiological surveys to detect chronic Chagas disease patients, which is the best diagnostic strategy*? It is recommended to employ ELISA or immunochromatography tests (ICT) for epidemiological studies of CD.

This recommendation is strong, because high confidence exists that ELISA or ICT, as single assays, are easily applicable in this epidemiological studies.

(iii) *Which is the best diagnostic method for blood bank screening?*

It is recommended to use highly sensitive ELISA kits or chemiluminescent magnetic immunoassays (CMIA). The quality of the evidence for this strong recommendation is considered high/moderate.

(iv) *In patients with suspicion of acute T. cruzi infection (congenital or recently acquired), which diagnostic methods are the most useful?*

In these cases, PAHO recommends the use of direct parasitological tests and eventual serological follow-up (in congenital infection after eight months of age and in infections caused from other transmission routes, seroconversion should be searched). The quality of the evidence for this recommendation is moderate.

**Knowledge gaps and diagnostic challenges**

Further improvements on diagnostic algorithms and methods, as well as expanded access to them are still necessary to be able to control CD. In epidemiological surveys and diagnosis of chronically infected subjects, it is expected that RDTs will widen access to diagnosis in large areas where well-equipped laboratories and qualified personnel are not commonly found.^98^ ^,^ ^102^

Easier and more rapid methods of extraction of nucleic acids are also still required for POC molecular diagnostic purposes. A 3D printer nucleic acids extraction-inspired machine has been devised to be coupled to LAMP,^108^ ^,^ ^109^ nicely complementing the remarkable POC characteristics of this isothermal amplification technology capable of yielding high sensitivity and specificity performance in resource-limited laboratories.^59^ Indeed, a recent pilot study in Yacuiba, Bolivia, showed that the so called PrinterLab LAMP duo strategy was able to detect congenitally infected neonates who were not identified by means of the current parasitological method.^108^

Field studies are needed to establish the potential of POC strategies for diagnosis of acute disease including congenital transmission. In this regard, accuracy of molecular detection of the infection from cord blood samples for an early diagnosis of congenital CD needs to be re-assessed. It is yet unclear what the rate of false positive results is when using this sample source, as it might be likely contaminated with parasite DNA from maternal blood and thus lead to false positive detections.^54^ ^,^ ^110^ ^,^ ^111^

On the other side, since several studies reported the use of dried blood on filter paper both for serological diagnosis^112^ ^,^ ^113^ and to detect parasitic DNA,^114^ exploring the use of dried blood spots, like Whatman 903 cards currently employed for neonatal screening of genetic diseases^59^ or FTA cards,^115^ ^,^ ^116^ would be very interesting to support molecular diagnosis assays. Beyond PCR and LAMP, other amplification technologies are being investigated, such as recombinase polymerase amplification (RPA). This isothermal reaction needs lower amplification temperature and shorter amplification times than LAMP, plus it has exhibited good performance in comparison to PCR in samples from domestic reservoirs in Mexico.^117^ Its combination to a modified 3D-printer low-cost DNA isolation system has been explored,^118^ so it would be worthwhile to evaluate this approach for *T. cruzi* diagnosis purposes.

## References

[1] (2006). No authors listed Chagas' disease - an epidemic that can no longer be ignored. Lancet.

[2] Garcia MN, Burroughs H, Gorchakov R, Gunter SM, Dumonteil E, Murray KO (2017). Molecular identification and genotyping of Trypanosoma cruzi DNA in autochthonous Chagas disease patients from Texas, USA. Infect Genet Evol.

[3] Schmunis GA, Yadon ZE (2010). Chagas disease a Latin American health problem becoming a world health problem. Acta Trop.

[4] Carlier Y, Altcheh J, Angheben A, Freilij H, Luquetti AO, Schijman AG (2019). Congenital Chagas disease updated recommendations for prevention, diagnosis, treatment, and follow-up of newborns and siblings, girls, women of childbearing age, and pregnant women. PLoS Negl Trop Dis.

[5] WHO - World Health Organization (2015). Chagas disease in Latin America: an epidemiological update based on 2010 estimates.

[6] Picado A, Cruz I, Redard-Jacot M, Schijman AG, Torrico F, Sosa-Estani S (2018). The burden of congenital Chagas disease and implementation of molecular diagnostic tools in Latin America. BMJ Glob Health.

[7] Howard EJ, Xiong X, Carlier Y, Sosa-Estani S, Buekens P (2014). Frequency of the congenital transmission of Trypanosoma cruzi a systematic review and meta-analysis. BJOG.

[8] Messenger LA, Gilman RH, Verastegui M, Galdos-Cardenas G, Sanchez G, Valencia E (2017). Towards improving early diagnosis of congenital Chagas disease in an endemic setting. Clin Infect Dis.

[9] Alarcon de Noya B.Diaz-Bello Z.Colmenares C.Ruiz-Guevara R.Mauriello L.Zavala-Jaspe R (2010). Large urban outbreak of orally acquired acute Chagas disease at a school in Caracas, Venezuela. J Infect Dis.

[10] Shikanai-Yasuda MA, Carvalho NB (2012). Oral transmission of Chagas disease. Clin Infect Dis.

[11] Santos EF, Silva ÂAO, Leony LM, Freitas NEM, Daltro RT, Regis-Silva CG (2020). Acute Chagas disease in Brazil from 2001 to 2018: a nationwide spatiotemporal analysis. PLoS Negl Trop Dis.

[12] Luquetti AO Schmuñis GA. In Telleria J, Tibayrenc M orgs (2017). American trypanosomiasis. Chagas disease. One hundred years of research. Diagnosis of Trypanosoma cruzi infection. Elsevier.

[13] Rassi A, Rassi A, Marin-Neto JA (2010). Chagas disease. Lancet.

[14] Alonso-Padilla J, Gallego M, Schijman AG, Gascon J (2017). Molecular diagnostics for Chagas disease up to date and novel methodologies. Expert Rev Mol Diagn.

[15] Egüez KE, Alonso-Padilla J, Terán C, Chipana Z, García W, Torrico F (2017). Rapid diagnostic tests duo as alternative to conventional serological assays for conclusive Chagas disease diagnosis. PLoS Negl Trop Dis.

[16] Tibayrenc M, Ward P, Moya A, Ayala FJ (1986). Natural populations of Trypanosoma cruzi, the agent of Chagas disease, have a complex multiclonal structure. Proc Natl Acad Sci USA.

[17] Sturm NR, Campbell DA (2010). Alternative lifestyles the population structure of Trypanosoma cruzi. Acta Trop.

[18] Macedo AM, Machado CR, Oliveira RP, Pena SDJ (2004). Trypanosoma cruzi genetic structure of populations and relevance of genetic variability to the pathogenesis of Chagas disease. Mem Inst Oswaldo Cruz.

[19] Miles MA, Llewellyn MS, Lewis MD, Yeo M, Baleela R, Fitzpatrick S (2009). The molecular epidemiology and phylogeography of Trypanosoma cruzi and parallel research on Leishmania looking back and to the future. Parasitology.

[20] Zingales B, Miles MA, Campbell DA, Tibayrenc M, Macedo AM, Teixeira MM (2012). The revised Trypanosoma cruzi subspecific nomenclature rationale, epidemiological relevance and research applications. Infect Genet Evol.

[21] Brenière SF, Waleckx E, Barnabé C (2016). Over six thousand Trypanosoma cruzi strains classified into discrete typing units (DTUs) attempt at an inventory. PLoS Negl Trop Dis.

[22] Lima L, Espinosa-Álvarez O, Ortiz PA, Trejo-Varón JA, Carranza JC, Pinto CM (2015). Genetic diversity of Trypanosoma cruzi in bats, and multilocus phylogenetic and phylogeographical analyses supporting Tcbat as an independent DTU (discrete typing unit). Acta Trop.

[23] Ramírez JD, Hernández C, Montilla M, Zambrano P, Flórez AC, Parra E (2014). First report of human Trypanosoma cruzi infection attributed to TcBat genotype. Zoonoses Public Health.

[24] Lewis MD, Llewellyn MS, Gaunt MW, Yeo M, Carrasco HJ, Miles MA (2009). Flow cytometric analysis and microsatellite genotyping reveal extensive DNA content variation in Trypanosoma cruzi populations and expose contrasts between natural and experimental hybrids. Int J Parasitol.

[25] Burgos JM, Begher S, Silva HM, Bisio M, Duffy T, Levin MJ (2008). Molecular identification of Trypanosoma cruzi I tropism for central nervous system in Chagas reactivation due to AIDS. Am J Trop Med Hyg.

[26] Burgos JM, Diez M, Vigliano C, Bisio M, Risso M, Duffy T (2010). Molecular identification of Trypanosoma cruzi discrete typing units in end-stage chronic Chagas heart disease and reactivation after heart transplantation. Clin Infect Dis.

[27] Souza RT, Lima FM, Barros RM, Cortez DR, Santos MF, Cordero EM (2011). Genome size, karyotype polymorphism and chromosomal evolution in Trypanosoma cruzi. PLoS One.

[28] Vargas N, Pedroso A, Zingales B (2004). Chromosomal polymorphism, gene synteny and genome size in T cruzi I and T. cruzi II groups. Mol Biochem Parasitol.

[29] OPS Guía para el diagnóstico y el tratamiento de la enfermedad de Chagas. http://iris.paho.org/xmlui/handle/10665.2/49653.

[30] Luquetti AO, Passos ADC, Silveira AC, Ferrerira AW, Macedo V, Prata AR (2011). O inquérito nacional de soroprevalência de avaliação do controle da doença de Chagas no Brasil (2001-2008). Rev Soc Bras Med Trop.

[31] Mendicino D, Colussi C, Moretti E (2019). Simultaneous use of two rapid diagnostic tests for the diagnosis of Chagas disease. Trop Doct.

[32] Lozano D, Rojas L, Méndez S, Casellas A, Sanz S, Ortiz L (2019). Use of rapid diagnostic tests (RDTs) for conclusive diagnosis of chronic Chagas disease - field implementation in the Bolivian Chaco region. PLoS Negl Trop Dis.

[33] Pérez-Molina JA, Molina I (2018). Chagas disease. Lancet.

[34] Luquetti AO, Andrade AZ, Barral-Neto M (2000). Trypanosoma cruzi e doenca de Chagas. Koogan.

[35] Strout RG (1962). A method for concentrating hemoflagellates. J Parasit.

[36] Santos AH, Silva IG, Rassi A (1995). Estudo comparativo entre o xenodiagnóstico natural e o artificial em chagásicos crônicos. Rev Soc Bras Med Trop.

[37] Castro AM, Luquetti AO, Rassi A, Chiari E, Galvão LM (2006). Detection of parasitaemia profiles by blood culture after treatment of human chronic Trypanosoma cruzi infection. Parasitol Res.

[38] Oliveira EC, Stefani MM, Luquetti AO, Vêncio EF, Moreira MA, Souza C (1993). Trypanosoma cruzi and experimental Chagas' disease characterization of a stock isolated from a patient with associated digestive and cardiac form. Rev Soc Bras Med Trop.

[39] Cerisola JA, Rohwedder RW, Del Prado CE (1971). [Yield of xenodiagnosis in human chronic Chagas' infection using nymphs of different species of triatomid bugs]. Bol Chil Parasitol.

[40] Affranchino JL, Ibañez CF, Luquetti AO, Rassi A, Reyes MB, Macina RA (1989). Identification of a Trypanosoma cruzi antigen that is shed during the acute phase of Chagas' disease. Mol Biochem Parasitol.

[41] Cabral HR (1983). Rheumatoid factors and Chagas' disease. Science.

[42] Carlier Y, Truyens C (2015). Congenital Chagas disease as an ecological model of interactions between Trypanosoma cruzi parasites, pregnant women, placenta and fetuses. Acta Trop.

[43] Torrico F, Alonso-Vega C, Suarez E, Rodriguez P, Torrico MC, Dramaix M (2004). Maternal Trypanosoma cruzi infection, pregnancy outcome, morbidity, and mortality of congenitally infected and non-infected newborns in Bolivia. Am J Trop Med Hyg.

[44] Liempi A, Castillo C, Carrillo I, Muñoz L, Droguett D, Galanti N (2016). A local innate immune response against Trypanosoma cruzi in the human placenta the epithelial turnover of the trophoblast. Microb Pathog.

[45] Antinori S, Galimberti L, Bianco R, Grande R, Galli M, Corbellino M (2017). Chagas disease in Europe a review for the internist in the globalized world. Eur J Intern Med.

[46] Requena-Méndez A, Albajar-Viñas P, Angheben A, Chiodini P, Gascón J, Muñoz J (2014). Chagas Disease COHEMI Working Group Health policies to control Chagas disease transmission in European countries. PLoS Negl Trop Dis.

[47] Cardoso EJ, Valdéz GC, Campos AC, de la Luz Sanchez R.Mendoza CR.Hernández AP (2012). Maternal fetal transmission of Trypanosoma cruzi a problem of public health little studied in Mexico. Exp Parasitol.

[48] Freilij H, Altcheh J (1994). Chagas congénito. Doyma.

[49] Bua J, Volta BJ, Perrone AE, Scollo K, Velázquez EB, Ruiz AM (2013). How to improve the early diagnosis of Trypanosoma cruzi infection relationship between validated conventional diagnosis and quantitative DNA amplification in congenitally infected children. PLoS Negl Trop Dis.

[50] Schijman AG, Altcheh J, Burgos JM, Biancardi M, Bisio M, Levin MJ (2003). Aetiological treatment of congenital Chagas' disease diagnosed and monitored by the polymerase chain reaction. J Antimicrob Chemother.

[51] Mora MC, Sanchez-Negrette O, Marco D, Barrio A, Ciaccio M, Segura MA (2005). Early diagnosis of congenital Trypanosoma cruzi infection using PCR, hemoculture, and capillary concentration, as compared with delayed serology. J Parasitol.

[52] Cura CI, Ramírez JC, Rodríguez M, Lopez-Albízu C, Irazu L, Scollo K (2017). Comparative study and analytical verification of PCR methods for the diagnosis of congenital Chagas disease. J Mol Diagn.

[53] Besuschio SA, Murcia ML, Benatar AF, Monnerat S, Cruz I, Picado A (2017). Analytical sensitivity and specificity of a loop-mediated isothermal amplification (LAMP) kit prototype for detection of Trypanosoma cruzi DNA in human blood samples. PLoS Negl Trop Dis.

[54] Benatar AF, Sosa-Estani S, Rojkin F, Schijman AG (2017). Validation of a real time PCR kit prototype for early diagnosis of congenital Chagas disease in a multicenter field study. Medicina.

[55] Bua J, Volta BJ, Velazquez EB, Ruiz AM, Rissio AM, Cardoni RL (2012). Vertical transmission of Trypanosoma cruzi infection quantification of parasite burden in mothers and their children by parasite DNA amplification. Trans R Soc Trop Med Hyg.

[56] Mori Y, Nagamine K, Tomita N, Notomi T (2001). Detection of loop-mediated isothermal amplification reaction by turbidity derived frm magnesium pyrophosphate formation. Biochem Biophys Res Commun.

[57] Notomi T, Okayama H, Masubuchi H, Yonekawa T, Watanabe K, Amino N (2000). Loop-mediated isothermal amplification of DNA. Nucleic Acids Res.

[58] Rivero R, Bisio M, Velázquez EB, Esteva MI, Scollo K, González NL (2017). Rapid detection of Trypanosoma cruzi by colorimetric loop-mediated isothermal amplification (LAMP) a potential novel tool for the detection of congenital Chagas infection. Diagn Microbiol Infect Dis.

[59] Besuschio SA, Picado A, Calderon-Muñoz A, Wehrendt DP, Fernández M, Benatar A (2020). Trypanosoma cruzi Loop mediated isothermal amplification (Trypanosoma cruzi Loopamp(tm)) kit for detection of congenital, acute or Chagas disease reactivation. PLoS Negl Trop Dis.

[60] Abras A, Ballart C, Llovet T, Roig C, Gutiérrez C, Tebar S (2018). Introducing automation to the molecular diagnosis of Trypanosoma cruzi infection a comparative study of sample treatments, DNA extraction methods and real-time PCR assays. PLoS One.

[61] Seiringer P, Pritsch M, Flores-Chávez M, Marchisio E, Helfrich K, Mengele C (2017). Comparison of four PCR methods for efficient detection of Trypanosoma cruzi in routine diagnostics. Diagn Microbiol Infect Dis.

[62] Besuschio SA, Wehrendt DP, Kuhn H, Longhi SA, Rottengatter K, Schijman AG (2019). Towards the use of qualitative real time PCR as a routine tool for Neglected Tropical Diseases: evaluation of a commercial kit for detection of Trypanosoma cruzi DNA. CAM.

[63] Volta BJ, Russomando G, Bustos PL, Scollo K, De Rissio AM, Sánchez Z (2015). Diagnosis of congenital Trypanosoma cruzi infection a serologic test using shed acute phase antigen (SAPA) in mother-child binomial samples. Acta Trop.

[64] Umezawa ES, Nascimento MS, Kesper N, Coura JR, Borges-Pereira J, Junqueira AC (1996). Immunoblot assay using excreted-secreted antigens of Trypanosoma cruzi in serodiagnosis of congenital, acute, and chronic Chagas' disease. J Clin Microbiol.

[65] Castro-Sesquén YE, Tinajeros F, Bern C, Galdos-Cardenas G, Malaga ES, Valencia Ayala E (2020). The IgM-SAPA-test for the early diagnosis of congenital Chagas disease in the time of the elimination goal of mother-to-child transmission. Clin Infect Dis.

[66] Castro-Sesquen YE, Gilman RH, Galdos-Cardenas G, Ferrufino L, Sánchez G, Valencia Ayala E (2014). Use of a novel Chagas urine nanoparticle test (Chunap) for diagnosis of congenital Chagas disease. PLoS Negl Trop Dis.

[67] Alarcón de Noya BA.Díaz-Bello Z.Colmenares C.Ruiz-Guevara R.Mauriello L.Muñoz-Calderón A (2015). Update on oral Chagas disease outbreaks in Venezuela epidemiological, clinical and diagnostic approaches. Mem Inst Oswaldo Cruz.

[68] Ramírez JD, Montilla M, Cucunubá ZM, Floréz AC, Zambrano P, Guhl F (2013). Molecular epidemiology of human oral Chagas disease outbreaks in Colombia. PLoS Negl Trop Dis.

[69] Blanchet D, Brenière SF, Schijman AG, Bisio M, Simon S, Véron V (2014). First report of a family outbreak of Chagas disease in French Guiana and posttreatment follow-up. Infect Genet Evol.

[70] Alarcón de Noya B.Colmenares C.Díaz-Bello Z.Ruiz-Guevara R.Medina K.Muñoz-Calderón A (2016). Orally-transmitted Chagas disease epidemiological, clinical, serological and molecular outcomes of a school microepidemic in Chichiriviche de la Costa, Venezuela. Parasit Epidemiol Control.

[71] Muñoz-Calderón A, Díaz-Bello Z, Ramírez JL, Noya O, Alarcón de Noya B (2019). Nifurtimox response of Trypanosoma cruzi isolates from an outbreak of Chagas disease in Caracas, Venezuela. Vector Borne Dis.

[72] WHO - World Health Organization (2014). Blood donor counselling: implementation guidelines.

[73] Angheben A, Boix L, Buonfrate D, Gobbi F, Bisoffi Z, Pupella S (2015). Chagas disease and transfusion medicine a perspective from non-endemic countries. Blood Transfus.

[74] Bern C (2012). Chagas disease in the immunosuppressed host. Curr Opin Infect Dis.

[75] Cura CI, Lattes R, Nagel C, Gimenez MJ, Blanes M, Calabuig E (2013). Early molecular diagnosis of acute Chagas disease after transplantation with organs from Trypanosoma cruzi-infected donors. Am J Transplant.

[76] Perez-Molina JA, Rodriguez-Guardado A, Soriano A, Pinazo MJ, Carrilero B, García-Rodríguez M (2011). Guidelines on the treatment of chronic coinfection by Trypanosoma cruzi and HIV outside endemic areas. HIV Clin Trials.

[77] Almeida EA, Ramos-Junior AN, Correia D, Shikanai-Yasuda MA (2011). Co-infection Trypanosoma cruzi/HIV systematic review (1980-2010). Rev Soc Bras Med Trop.

[78] da Costa PA, Segatto M, Durso DF, Moreira WJC, Junqueira LL, de Castilho FM (2017). Early polymerase chain reaction detection of Chagas disease reactivation in heart transplant patients. J Heart Lung Transplant.

[79] Diez M, Favaloro L, Bertolotti A, Burgos JM, Vigliano C, Lastra MP (2007). Usefulness of PCR strategies for early diagnosis of Chagas' disease reactivation and treatment follow-up in heart transplantation. Am J Transplant.

[80] Burgos JM, Begher SB, Freitas JM, Bisio M, Duffy T, Altcheh J (2005). Molecular diagnosis and typing of Trypanosoma cruzi populations and lineages in cerebral Chagas disease in a patient with AIDS. Am J Trop Med Hyg.

[81] Castro-Sesquen YE, Gilman RH, Mejia C, Clark DE, Choi J, Reimer-McAtee MJ (2016). Use of a Chagas urine nanoparticle test (Chunap) to correlate with parasitemia levels in T cruzi/HIV co-infected patients. PLoS Negl Trop Dis.

[82] Schijman AG, Bisio M, Orellana L, Sued M, Duffy T, Mejia Jaramillo AM (2011). International study to evaluate PCR methods for detection of Trypanosoma cruzi DNA in blood samples from Chagas disease patients. PLoS Negl Trop Dis.

[83] Brasil PE, De Castro L, Hasslocher-Moreno AM, Sangenis LH, Braga JU (2010). ELISA versus PCR for diagnosis of chronic Chagas disease systematic review and meta-analysis. BMC Infect Dis.

[84] Hernández C, Cucunubá Z, Flórez C, Olivera M, Valencia-Hernandez CA, Zambrano P (2016). Molecular diagnosis of Chagas disease in Colombia parasitic loads and discrete typing units in patients from acute and chronic phases. PLoS Negl Trop Dis.

[85] Ramírez JC, Cura CI, Moreira OC, Lages-Silva E, Juiz N, Velázquez E (2015). Analytical validation of quantitative real-time PCR methods for quantification of Trypanosoma cruzi DNA in blood samples from Chagas disease patients. J Mol Diagn.

[86] Umezawa ES, Bastos SF, Coura JR, Levin MJ, Gonzalez A, Rangel-Aldao R (2003). An improved serodiagnostic test for Chagas' disease employing a mixture of Trypanosoma cruzi recombinant antigens. Transfusion.

[87] Mucci J, Carmona SJ, Volcovich R, Altcheh J, Bracamonte E, Marco JD (2017). Next-generation ELISA diagnostic assay for Chagas disease based on the combination of short peptidic epitopes. PLoS Negl Trop Dis.

[88] Del-Rei RP, Leony LM, Celedon PAF, Zanchin NIT, Reis MGD, Gomes YM (2019). Detection of anti-Trypanosoma cruzi antibodies by chimeric antigens in chronic Chagas disease-individuals from endemic South American countries. PLoS One.

[89] Abras A, Gállego M, Llovet T, Tebar S, Herrero M, Berenguer P (2016). Serological diagnosis of chronic Chagas disease is it time for a change?. J Clin Microbiol.

[90] Almeida IC, Ferguson MA, Schenkman S, Travassos LR (1994). GPI-anchored glycoconjugates from Trypanosoma cruzi trypomastigotes are recognized by lytic anti-alpha-galactosyl antibodies isolated from patients with chronic Chagas' disease. Braz J Med Biol Res.

[91] Almeida IC, Covas DT, Soussumi LM, Travassos LR (1997). A highly sensitive and specific chemiluminescent enzyme-linked immunosorbent assay for diagnosis of active Trypanosoma cruzi infection. Transfusion.

[92] Izquierdo L, Marques AF, Gállego M, Sanz S, Tebar S, Riera C (2013). Evaluation of a chemiluminescent enzyme-linked immunosorbent assay for the diagnosis of Trypanosoma cruzi infection in a nonendemic setting. Mem Inst Oswaldo Cruz.

[93] Torrico F, Gascon J, Ortiz L, Alonso-Vega C, Pinazo MJ, Schijman A (2018). Treatment of adult chronic indeterminate Chagas disease with benznidazole and three E1224 dosing regimens a proof-of-concept, randomised, placebo-controlled trial. Lancet Infect Dis.

[94] Umezawa ES, Souza AI, Pinedo-Cancino V, Marcondes M, Marcili A, Camargo LM (2009). TESA-blot for the diagnosis of Chagas disease in dogs from co-endemic regions for Trypanosoma cruzi, Trypanosoma evansi and Leishmania chagasi. Acta Trop.

[95] Shah DO, Chang C-D, Cheng KY, Salbilla VA, Adya N, Marchlewicz BA (2010). Comparison of the analytic sensitivities of a recombinant immunoblot assay and the radioimmune precipitation assay for the detection of antibodies to Trypanosoma cruzi in patients with Chagas disease. Diagn Microbiol Infect Dis.

[96] Granjon E, Dichtel-Danjoy M-L.Saba E.Sabino E.Campos de Oliveira L.Zrein M (2016). Development of a novel multiplex immunoassay multi-cruzi for the serological confirmation of Chagas disease. PLoS Negl Trop Dis.

[97] OPS (2020). Síntesis de evidencia: Guía para el diagnóstico y el tratamiento de la enfermedad de Chagas. Rev Panam Salud Publica.

[98] Luquetti AO, Ponce C, Ponce E, Esfandiari J, Schijman A, Revollo S (2003). Chagas disease diagnosis a multicentric evaluation of Chagas stat-Pak, a rapid immunochromatographic assay with recombinant proteins of Trypanosoma cruzi. J Diagn Microbiol Infect Dis.

[99] Sánchez-Camargo CL, Albajar-Vinas P, Wilkins PP, Nieto J, Leiby DA, Paris L (2014). Comparative evaluation of 11 commercialized rapid diagnostic tests for detecting Trypanosoma cruzi antibodies in serum banks in areas of endemicity and non endemicity. J Clin Microbiol.

[100] WHO (2012). WHO Technical Report Series Nº 905. Control of Chagas disease. Second report of the WHO Expert Committee. World Health Organization.

[101] Verani JR, Seitz A, Gilman RH, LaFuente C, Galdos-Cardenas G, Kawai V (2009). Geographic variation in the sensitivity of recombinant antigen-based rapid tests for chronic Trypanosoma cruzi infection. Am J Trop Med Hyg.

[102] Shah V, Ferrufino L, Gilman RH, Ramirez M, Saenza E, Malaga E (2014). Field evaluation of the InBios Chagas detect plus rapid test in serum and whole-blood specimens in Bolivia. Clin Vaccine Immunol.

[103] Angheben A, Buonfrate D, Cruciani M, Jackson Y, Alonso-Padilla J, Gascon J (2019). Rapid immunochromatographic tests for the diagnosis of chronic Chagas disease in at-risk populations a systematic review and meta-analysis. PLoS Negl Trop Dis.

[104] Teixeira AR, Teixeira MG (1995). [Delayed hypersensitivity to Trypanosoma cruzi antigen. III - Sensitivity of the skin test with T12E antigen in the diagnosis of Chagas disease in hospitalized patients]. Rev Soc Bras Med Trop.

[105] Málaga-Machaca ES, Romero-Ramirez A, Gilman RH, Astupiña-Figueroa S, Angulo N, Florentini A (2017). Polyclonal antibodies for the detection of Trypanosoma cruzi circulating antigens. PLoS Negl Trop Dis.

[106] OPS - Organización Panamericana de la Salud (2018). Guía para el diagnóstico y el tratamiento de la enfermedad de Chagas.

[107] Guyatt GH, Oxman AD, Kunz R, Atkins D, Brozek J, Vist G (2011). GRADE guidelines 2. Framing the question and deciding on important outcomes. J Clin Epidemiol.

[108] Wehrendt DP, Alonso-Padilla J, Liu B, Panozo LR, Nina SR, Pinto L (2020). Development and evaluation of a 3D Printer-based DNA extraction method coupled to loop mediated isothermal amplification (LAMP) for point-of-care diagnosis of congenital Chagas disease in endemic regions. J Mol Diagn.

[109] Chan K, Coen M, Hardick J, Gaydos CA, Wong KY, Smith C (2016). Low-cost 3D printers enable high-quality and automated sample preparation and molecular detection. PLoS One.

[110] Carlier Y, Truyens C (2017). In Telleria J, Tibayrenc M, editors. Maternal-fetal transmission of Trypanosoma cruzi. American trypanosomiasis-Chagas disease. One hundred years of research. 2nd ed. Elsevier.

[111] Buekens P, Cafferata ML, Alger J, Althabe F, Belizán JM, Bustamante N (2018). Congenital transmission of Trypanosoma cruzi in Argentina, Honduras, and Mexico an observational prospective study. Am J Trop Med Hyg.

[112] Zicker F, Smith PG, Luquetti AO, Oliveira OS (1990). Mass screening for Trypanosoma cruzi infections using the immunofluorescence, ELISA and haemagglutination tests on serum samples and on blood eluates from filter-paper. Bull World Health Organ.

[113] Holguín A, Norman F, Martín L, Mateos ML, Chacón J, López-Vélez R (2013). Dried blood as an alternative to plasma or serum for Trypanosoma cruzi IgG detection in screening programs. Clin Vaccine Immunol.

[114] Sánchez AG, Alvarellos E, Kohout I, Schulz DGR, Cordeiro E, Caeiro JP (2016). Detection of Trypanosoma cruzi and treatment monitoring by PCR from dried blood spot samples in children. Rev Fac Cien Med Univ Nac Cordoba.

[115] Ahmed HA, MacLeod ET, Hide G, Welburn SC, Picozzi K (2011). The best practice for preparation of samples from FTA(r)cards for diagnosis of blood borne infections using African trypanosomes as a model system. Parasit Vectors.

[116] Hashimoto M, Bando M, Kido JI, Yokota K, Mita T, Kajimoto K (2019). Nucleic acid purification from dried blood spot on FTA Elute Card provides template for polymerase chain reaction for highly sensitive Plasmodium detection. Parasitol Int.

[117] Jimenez-Coello M, Shelite T, Castellanos-Gonzalez A, Saldarriaga O, Rivero R, Ortega-Pacheco A (2018). Efficacy of recombinase polymerase amplification to diagnose Trypanosoma cruzi infection in dogs with cardiac alterations from an endemic area of Mexico. Vector Borne Zoonotic Dis.

[118] Chan K, Wong PY, Parikh C, Wong S (2018). Moving toward rapid and low-cost point-of-care molecular diagnostics with a repurposed 3D printer and RPA. Anal Biochem.

